# Global trends in disease burdens attributable to ambient and household air pollution: a comparative study of ten populous countries

**DOI:** 10.3389/fpubh.2025.1629616

**Published:** 2025-07-31

**Authors:** Jing Feng, Yannan Wang, Xianghua Meng, Yue Fang

**Affiliations:** ^1^Department of Rehabilitation Medicine, General Hospital of Northern Theater Command, Shenyang, China; ^2^Cadre’s Ward, General Hospital of Northern Theater Command, Shenyang, China

**Keywords:** ambient particulate matter, household air pollution, disability-adjusted life years, socio-demographic index, disease burden, public health

## Abstract

**Objectives:**

To comparatively assess the disease burden attributable to ambient particulate matter (APM) and household air pollution (HAP) across the ten most populous countries between 1990 and 2021.

**Methods:**

Data on disability-adjusted life years (DALYs) and age-standardized DALY rates (ASDRs) attributable to APM and HAP were obtained from the Global Burden of Disease Study 2021. Estimated annual percentage change (EAPC) was used to evaluate temporal trends. Quadratic regression models were applied to examine associations between socio-demographic index (SDI) and disease burden.

**Results:**

From 1990 to 2021, global APM-related DALYs increased while ASDRs declined. In contrast, both DALYs and ASDRs attributable to HAP decreased markedly. Older adults experienced the highest APM-related burden, whereas children under five were most affected by HAP. Gender differences were also observed, with males generally showing higher burdens. SDI was strongly associated with HAP-related ASDRs (*R*^2^ = 0.88) but weakly associated with APM (*R*^2^ = 0.19).

**Conclusion:**

Significant disparities in air pollution-related disease burdens exist across countries and demographic groups. Clean energy policies, strengthened environmental regulations, and targeted interventions are essential to mitigate health impacts and advance global public health equity.

## 1 Introduction

Air pollution, particularly ambient particulate matter (APM) and household air pollution (HAP), represents a significant global public health challenge. APM, dominated by fine particles (PM2.5), is a critical environmental risk factor associated with severe health outcomes, including chronic respiratory diseases, cardiovascular diseases, and lung cancer (1, 2). HAP, primarily generated by combustion of solid fuels such as wood, coal, and biomass in inefficient stoves, remains an important cause of indoor air quality deterioration, disproportionately impacting populations in low- and middle-income countries (3, 4).

The Global Burden of Disease (GBD) study has extensively quantified the health impacts of these air pollutants, revealing substantial disease burdens in regions characterized by poor air quality and limited access to clean energy sources (5). Vulnerable populations, including children, the older adult, and women—who are often exposed to indoor pollutants during cooking and heating activities—bear a disproportionate share of this burden (6, 7).

The ten most populous countries—India, China, the United States, Indonesia, Pakistan, Brazil, Nigeria, Bangladesh, Russia, and Mexico—comprise a significant proportion of the global population, presenting diverse socioeconomic contexts and thus providing a critical framework for examining variations in disease burdens related to air pollution. While some nations have made notable progress in improving air quality through effective policy implementation and cleaner fuel adoption, others continue to encounter substantial challenges in reducing pollution exposures and associated health risks (8, 9).

This study aims to comparatively analyze the disease burden attributable to APM and HAP across these ten countries. Through assessing long-term trends, demographic disparities, and specific health outcomes linked to these environmental risk factors, we seek to highlight existing global health inequities and inform targeted public health interventions. Understanding regional variations in air pollution-related health impacts is crucial for achieving the United Nations’ Sustainable Development Goals (SDGs), particularly Goal 3 (Good Health and Well-being) and Goal 7 (Affordable and Clean Energy) (10, 11).

## 2 Methods

### 2.1 Data acquisition and sources

This observational study utilized data from the GBD 2021 study, which provides comprehensive global estimates of disease burdens attributed to various risk factors across 204 countries and territories (12). Specifically, we extracted disease burden estimates attributable to APM and HAP for the ten most populous countries (India, China, the United States, Indonesia, Pakistan, Brazil, Nigeria, Bangladesh, Russia, and Mexico) from 1990 to 2021. Although Ethiopia has recently surpassed Mexico in population size, Mexico was included to maintain consistency with the GBD 2021 dataset, which designates these ten countries as the most populous.

Data on disability-adjusted life years (DALYs), age-standardized DALY rates (ASDRs), and associated 95% uncertainty intervals (UIs) were publicly obtained through the Global Health Data Exchange query tool.^1^ DALYs represent the total health loss from both premature mortality and non-fatal health conditions, and are calculated as the sum of years of life lost (YLLs) and years lived with disability (YLDs) (13). ASDR account for differences in age structure across populations, allowing for fair comparisons between countries and over time.

To ensure focus and clarity, we included the top seven Level 3 causes of DALYs that are shared by both APM and HAP at the global level, according to GBD 2021 data. These diseases include Chronic Obstructive Pulmonary Disease (COPD), Ischemic Heart Disease (IHD), Stroke, Lower Respiratory Infections (LRI), Neonatal Disorders, Diabetes Mellitus (DM), and Tracheal, Bronchus, and Lung Cancer. By focusing on this common set of high-burden diseases, we ensured consistency and comparability in evaluating the impact of both risk factors, while simplifying the analysis by excluding numerous lower-burden causes.

The GBD study employs de-identified secondary data, and ethics approval was not required due to the public availability and anonymous nature of the dataset. Our study adheres to the Guidelines for Accurate and Transparent Health Estimates Reporting (GATHER).

### 2.2 Estimation framework

The GBD 2021 employs robust standardized methods to estimate the global burden of disease, using tools such as DisMod-MR 2.1 and the Cause of Death Ensemble model (CODEm) (13). DisMod-MR 2.1 is a Bayesian meta-regression tool used to ensure internal consistency across epidemiological parameters, including prevalence, incidence, remission, and excess mortality rates by age, sex, and location. For cause-specific mortality estimation, CODEm systematically evaluates an ensemble of statistical models and selects the most robust predictive model combinations based on out-of-sample predictive performance, providing cause-specific death estimates stratified by age, sex, year, and location.

Disease burden attributed to air pollution was estimated using a comparative risk assessment (CRA) approach (14). This method compares actual exposure distributions to theoretical minimum risk exposure levels (TMREL) to calculate population attributable fractions, which were subsequently applied to overall disease burdens to derive pollution-specific DALY estimates.

### 2.3 Definition of risk factors

APM refers to outdoor exposure to particulate matter with an aerodynamic diameter ≤2.5 μm (PM2.5), primarily from anthropogenic sources such as fossil fuel combustion, industrial emissions, vehicle exhaust, and biomass burning (14). Exposure levels were estimated using satellite-based aerosol optical depth data, ground-based monitoring stations, and chemical transport models.

HAP refers to indoor pollution from the combustion of solid fuels, including wood, crop residues, dung, charcoal, and coal, typically used for cooking and heating. Exposure estimation was based on national household surveys reporting the proportion of households using solid fuels as their primary energy source.

### 2.4 Socio-demographic index

The socio-demographic index (SDI) is a composite indicator developed by the GBD study researchers (15). It integrates three dimensions: total fertility rate under age 25, mean years of education for the population aged ≥15 years, and lag-distributed income per capita. SDI values range from 0 (lowest socio-demographic development) to 1 (highest socio-demographic development). SDI data were extracted from the GBD database and utilized to examine the relationship between socio-economic development and disease burdens attributed to air pollution.

### 2.5 Statistical analysis

To quantify temporal trends in the air pollution-attributable disease burden, the Estimated Annual Percentage Change (EAPC) was calculated (16). The EAPC was derived from linear regression analysis of the natural logarithm of the ASDR using the formula: $\text{EAPC}=(e^{\beta }-1)\times 100\%$ where β represents the regression coefficient. Trends were considered significant if the 95% confidence intervals (CI) did not include zero; a positive EAPC indicated an increasing trend, while a negative EAPC indicated a decreasing trend.

Quadratic regression analysis was performed to examine the non-linear association between the SDI and ASDR attributable to air pollution (17). Specifically, a second-order polynomial regression model was constructed: $y=a+\mathrm{\beta x}+\gamma x^{2}+\varepsilon$ where y represents the ASDR, χ represents the SDI, a is the intercept, β and γ are the regression coefficients, and ε is the error term. The significance of regression coefficients was assessed using t-tests, and the goodness-of-fit was evaluated using the coefficient of determination (R^2^). Statistical significance was defined as a *p*-value < 0.05.

All statistical analyses and visualizations were performed using R statistical software (version 4.4.1).

## 3 Results

### 3.1 Trends of disease burden from APM and HAP (1990–2021)

Between 1990 and 2021, the global disease burden attributable to APM increased significantly, with DALYs rising from 77459.73 thousand to 120004.67 thousand. In contrast, the global ASDR for APM declined from 1716.81 per 100,000 in 1990 to 1483.61 per 100,000 in 2021, indicating an EAPC of−0.28% (95% CI: −0.43% to −0.12%) (Table 1).

**Table 1:** DALYs and age-standardized DALY rates attributed to APM and HAP in 1990 and 2021, and their trends.

	Cases in 1990, thousands (95% UI)	Age-standardized rate per 100,000 population in 1990 (95% UI)	Cases in 2021, thousands (95% UI)	Age-standardized rate per 100,000 population in 2021 (95% UI)	EAPC (%) (95% CI)
Ambient particulate matter pollution					
Global	77459.73 (55118.40, 102684.11)	1716.81 (1240.07, 2235.75)	120004.67 (86560.33, 149810.19)	1483.61 (1069.48, 1869.55)	−0.28 (−0.43, −0.12)
India	11073.88 (5851.74, 18278.44)	1486.42 (819.57, 2404.90)	27408.37 (17694.22, 36250.43)	2382.38 (1528.11, 3149.89)	1.96 (1.55, 2.37)
China	12726.19 (6156.14, 22700.34)	1590.95 (768.20, 2818.49)	37805.87 (26280.46, 46518.70)	1970.10 (1373.00, 2423.21)	1.19 (0.85, 1.54)
United States	2901.45 (1292.99, 4773.39)	947.06 (433.81, 1537.76)	1201.10 (608.37, 1904.32)	224.24 (118.08, 345.47)	−5.06 (−5.45, −4.66)
Indonesia	1666.94 (711.07, 3089.68)	1203.60 (512.92, 2251.44)	3826.06 (2328.96, 5341.69)	1644.73 (1009.26, 2279.86)	0.72 (0.56, 0.87)
Pakistan	1977.35 (933.84, 3519.50)	1707.23 (840.51, 2984.66)	4399.13 (2290.88, 6814.14)	2501.60 (1296.20, 3839.21)	1.39 (1.04, 1.74)
Nigeria	3036.24 (1395.11, 4945.27)	2523.79 (1238.04, 3967.10)	4358.41 (2079.33, 7728.97)	1943.68 (986.42, 3259.99)	0.17 (−0.33, 0.67)
Brazil	1374.15 (539.17, 2359.84)	1282.39 (472.38, 2260.52)	1396.91 (828.75, 2016.70)	581.53 (356.77, 833.51)	−2.61 (−2.81, −2.42)
Bangladesh	1252.56 (571.34, 2284.45)	1133.93 (562.43, 2028.49)	1188.38 (635.86, 2029.27)	879.49 (469.26, 1492.67)	−0.77 (−1.17, −0.37)
Russia	4605.56 (2313.73, 6924.12)	2806.64 (1459.83, 4188.73)	1889.92 (1146.33, 2862.61)	826.57 (507.47, 1238.26)	−4.69 (−5.21, −4.17)
Mexico	1411.24 (800.41, 2049.61)	2153.90 (1230.34, 3058.91)	1125.57 (758.25, 1489.39)	918.36 (621.91, 1208.19)	−2.89 (−3.09, −2.69)
Household air pollution					
Global	211860.69 (154596.44, 265090.27)	4147.68 (3101.41, 5104.55)	111462.96 (75085.85, 163710.71)	1500.29 (1028.38, 2195.56)	−3.52 (−3.77, −3.27)
India	56268.40 (42437.80, 69208.62)	7261.66 (5740.88, 8585.29)	33523.36 (22090.87, 48262.49)	2966.21 (1971.31, 4256.39)	−2.82 (−3.05, −2.58)
China	53590.42 (40745.95, 64919.09)	6551.36 (5073.76, 7895.68)	8858.34 (1494.83, 27820.52)	466.10 (81.56, 1455.82)	−8.98 (−9.78, −8.18)
United States	1.96 (0.01, 13.77)	0.64 (0, 4.57)	0.78 (−0.03, 3)	0.14 (0, 0.54)	−5.14 (−5.29, −5.00)
Indonesia	6790.72 (5125.61, 8462.14)	4952.02 (3844.42, 6012.87)	2694.76 (1052.36, 5441.30)	1184.72 (462.53, 2363.92)	−4.32 (−4.96, −3.67)
Pakistan	7645.12 (5437.61, 9907.48)	6343.94 (4772.55, 7781.96)	6921.59 (4201.41, 9894.37)	3775.44 (2293.14, 5291.28)	−1.82 (−2.08, −1.56)
Nigeria	8477.70 (5071.53, 12292.64)	6929.22 (4362.79, 9482.67)	8434.47 (5020.97, 12788.85)	3426.03 (2058.90, 5050.15)	−2.64 (−2.91, −2.37)
Brazil	1897.84 (1143.04, 2953.03)	1666.87 (970.44, 2643.27)	296.17 (82.59, 804.79)	126.10 (36.33, 339.14)	−8.31 (−8.59, −8.03)
Bangladesh	11789.46 (8016.84, 14971.64)	10001.07 (7857.52, 11993.52)	5711.98 (4345.50, 7211.61)	4234.79 (3234.85, 5350.70)	−2.73 (−2.95, −2.50)
Russia	186.52 (19.82, 1025.69)	117.51 (13.31, 639.57)	28.01 (0.73, 217.92)	12.22 (0.32, 94.47)	−10.24 (−12.32, −8.12)
Mexico	431.85 (90.47, 1052.95)	611.98 (122.46, 1519.09)	178.29 (25.51, 640.73)	149.39 (21.75, 533.13)	−4.81 (−4.93, −4.69)

DALY, Disability-Adjusted Life Years; UI, uncertainty interval; EAPC, Estimated Annual Percentage Change; CI, confident interval.

**Figure 1:**
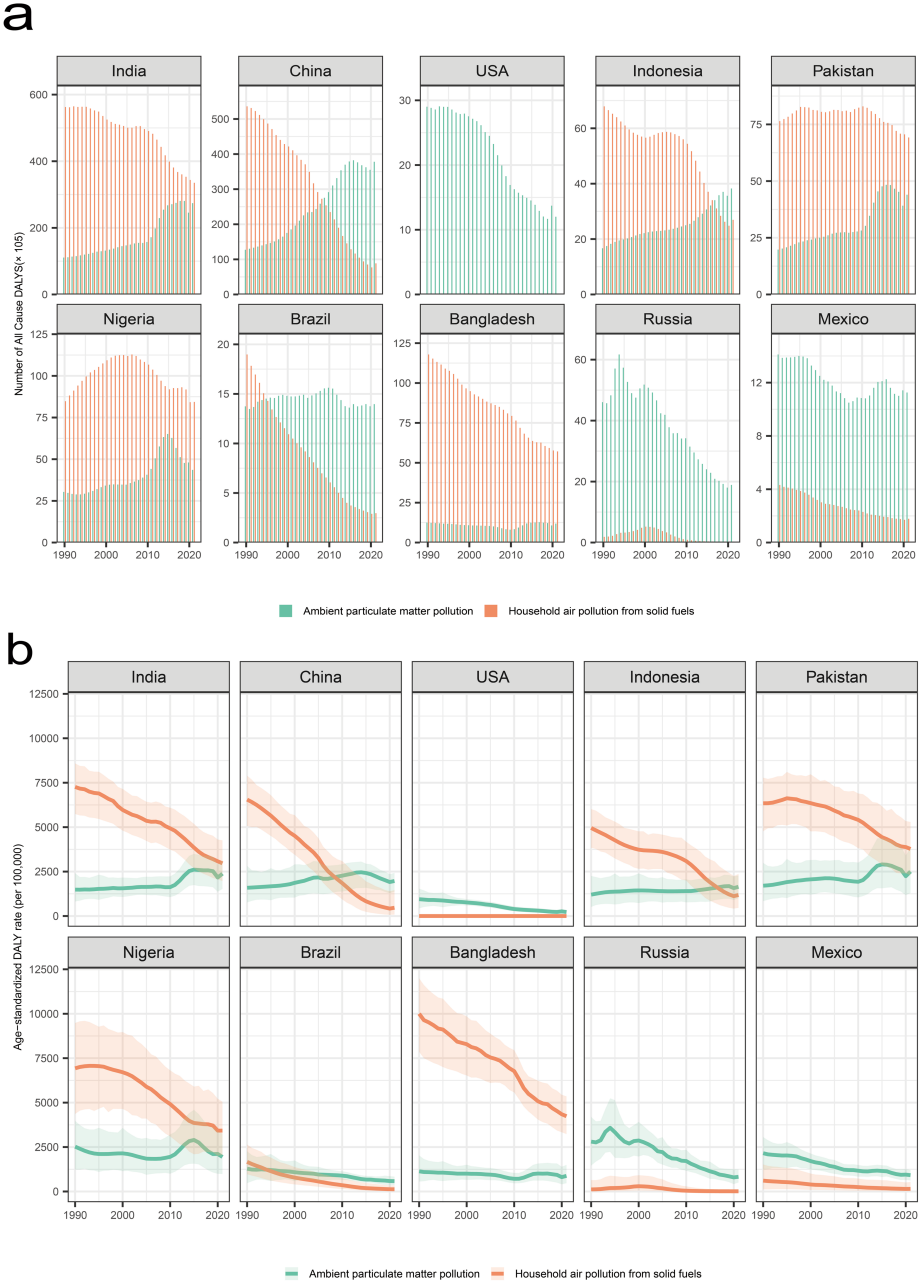
**(a)** Trends in all-cause disability-adjusted life years (DALYs) attributed to ambient particulate matter (APM) and household air pollution (HAP) across the ten most populous countries between 1990 and 2021; **(b)** Trends in age-standardized DALY rates attributed to APM and HAP in the same countries over the same period. DALY, disability-adjusted life year; APM, ambient particulate matter; HAP, household air pollution.

Among the ten most populous countries, China and India experienced the largest absolute increases in APM-related disease burden. In China, DALYs rose from 12726.19 thousand in 1990 to 37805.87 thousand in 2021, with ASDR increasing from 1590.95 to 1970.10 per 100,000, corresponding to a positive EAPC of 1.19% (95% CI: 0.85 to 1.54%). Similarly, India’s DALYs increased from 11073.88 thousand to 27408.37 thousand, while ASDR rose markedly from 1486.42 to 2382.38 per 100,000, with an EAPC of 1.96% (95% CI: 1.55 to 2.37%). Pakistan and Indonesia also exhibited substantial increases in APM-related DALYs, reaching 4399.13 thousand and 3826.06 thousand, respectively, by 2021, accompanied by corresponding ASDR increases. Nigeria demonstrated a modest increase in DALYs with an almost stable EAPC of 0.17% (95% CI: −0.33 to 0.67%). Conversely, the United States, Russia, Brazil, Bangladesh, and Mexico recorded significant declines in both absolute DALYs and ASDRs. Notably, in the United States, DALYs decreased from 2901.45 thousand in 1990 to 1201.10 thousand in 2021, while ASDR dropped from 947.06 to 224.24 per 100,000, corresponding to an EAPC of−5.06% (95% CI: −5.45% to −4.66%). Russia showed a similar trend, with DALYs declining from 4605.56 thousand to 1889.92 thousand and ASDR decreasing from 2806.64 to 826.57 per 100,000 (EAPC: −4.69, 95% CI: −5.21% to −4.17%). Brazil and Mexico also achieved notable reductions in ASDRs, with EAPCs of−2.61% (95% CI: −2.81% to −2.42%) and −2.89% (95% CI: −3.09% to −2.69%), respectively (Figure 1).

Regarding HAP, global DALYs decreased substantially from 211860.69 thousand in 1990 to 111462.96 thousand in 2021. Consistently, the global ASDR dropped from 4147.68 per 100,000 to 1500.29 per 100,000, corresponding to an EAPC of −3.52% (95% CI: −3.77% to −3.27%).

China exhibited the most significant reduction in DALYs attributed to HAP, declining from 53590.42 thousand in 1990 to 8858.34 thousand in 2021, with ASDR decreasing from 6551.36 to 466.10 per 100,000 (EAPC: −8.98, 95% CI: −9.78% to −8.18%). Although India continued to bear the highest burden, its DALYs dropped markedly from 56268.40 thousand to 33523.36 thousand, with ASDR falling from 7261.66 to 2966.21 per 100,000 (EAPC: −2.82, 95% CI: −3.05% to −2.58%). Similar decreasing trends were observed in Indonesia, Pakistan, Nigeria, Bangladesh, Brazil, Russia, and Mexico, with Brazil and Russia achieving the most pronounced reductions (EAPCs: −8.31% [95% CI: −8.59% to −8.03%] and −10.24% [95% CI: −12.32% to −8.12%], respectively).

### 3.2 Disease burden by age and gender

#### 3.2.1 Age group analysis

**Figure 2:**
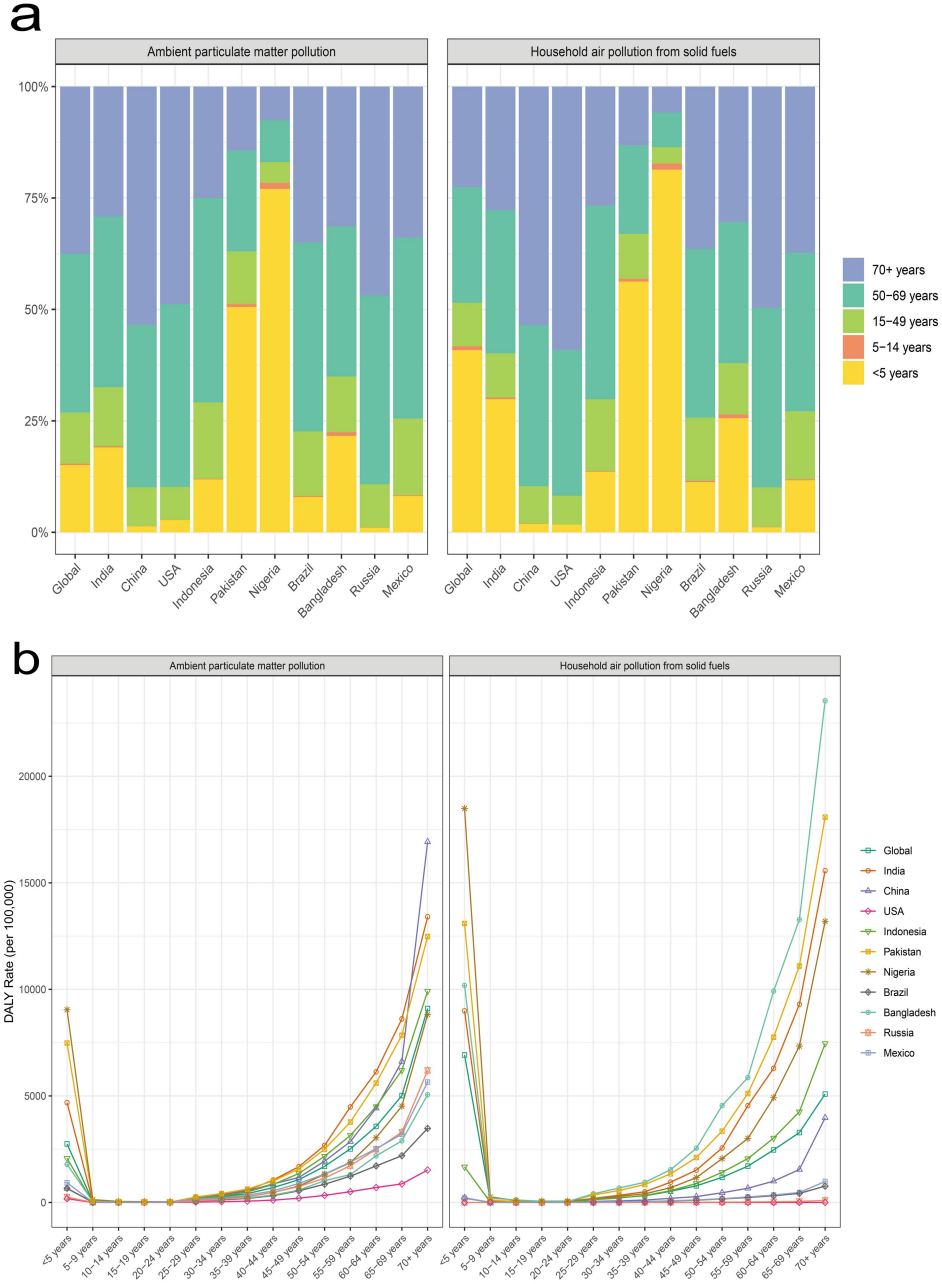
**(a)** Age group distribution of all-cause DALYs attributed to APM and HAP, globally and in the ten most populous countries in 2021; **(b)** Age-specific DALY rates attributed to APM and HAP, globally and in the ten most populous countries in 2021. DALY, disability-adjusted life year; APM, ambient particulate matter; HAP, household air pollution.

In 2021, the disease burden due to APM pollution was disproportionately higher in older populations, particularly those aged 70 years and above. Globally, this group had the highest DALY rate (9101.91 per 100,000) and accounted for 37.50% of the total APM-related burden. Similar age-related trends appeared distinctly in China (53.43%, DALY rate: 16930.09 per 100,000), India (29.15%, DALY rate: 13406.67 per 100,000), and Indonesia (25.00%, DALY rate: 9915.26 per 100,000). Conversely, children under 5 years had relatively lower global proportions (15.08%), although their DALY rates were notably high in countries like Nigeria (9045.96 per 100,000, 77.04%) and Pakistan (7482.86 per 100,000, 50.56%). Regarding HAP, young children under 5 years carried a notably high burden globally, with a DALY rate of 6923.59 per 100,000 and accounting for 40.88% of total DALYs. Particularly high burdens were observed in Nigeria (18487.18 per 100,000, 81.36%), Pakistan (13095.46 per 100,000, 56.24%), and India (8993.05 per 100,000, 29.87%). In contrast, the older adult (aged 70+) bore substantial burdens in countries with improved indoor air quality such as China (3974.22 per 100,000, 53.52%) and the United States (1.19 per 100,000, 59.10%) (Figure 2).

#### 3.2.2 Gender-specific analysis

Gender-Specific Analysis: For APM, the global age-standardized DALY rate in 2021 was 1871.56 per 100,000 for males and 1138.28 per 100,000 for females. Among the ten countries, India reported rates of 2930.98 (males) and 1871.56 (females); Pakistan, 2902.16 and 2067.39; China, 2604.27 and 1455.28; Indonesia, 2000.32 and 1314.48; and Nigeria, 2310.05 and 1608.02, respectively. The United States exhibited the lowest APM-related burden, with 274.83 for males and 179.66 for females per 100,000.

**Figure 3:**
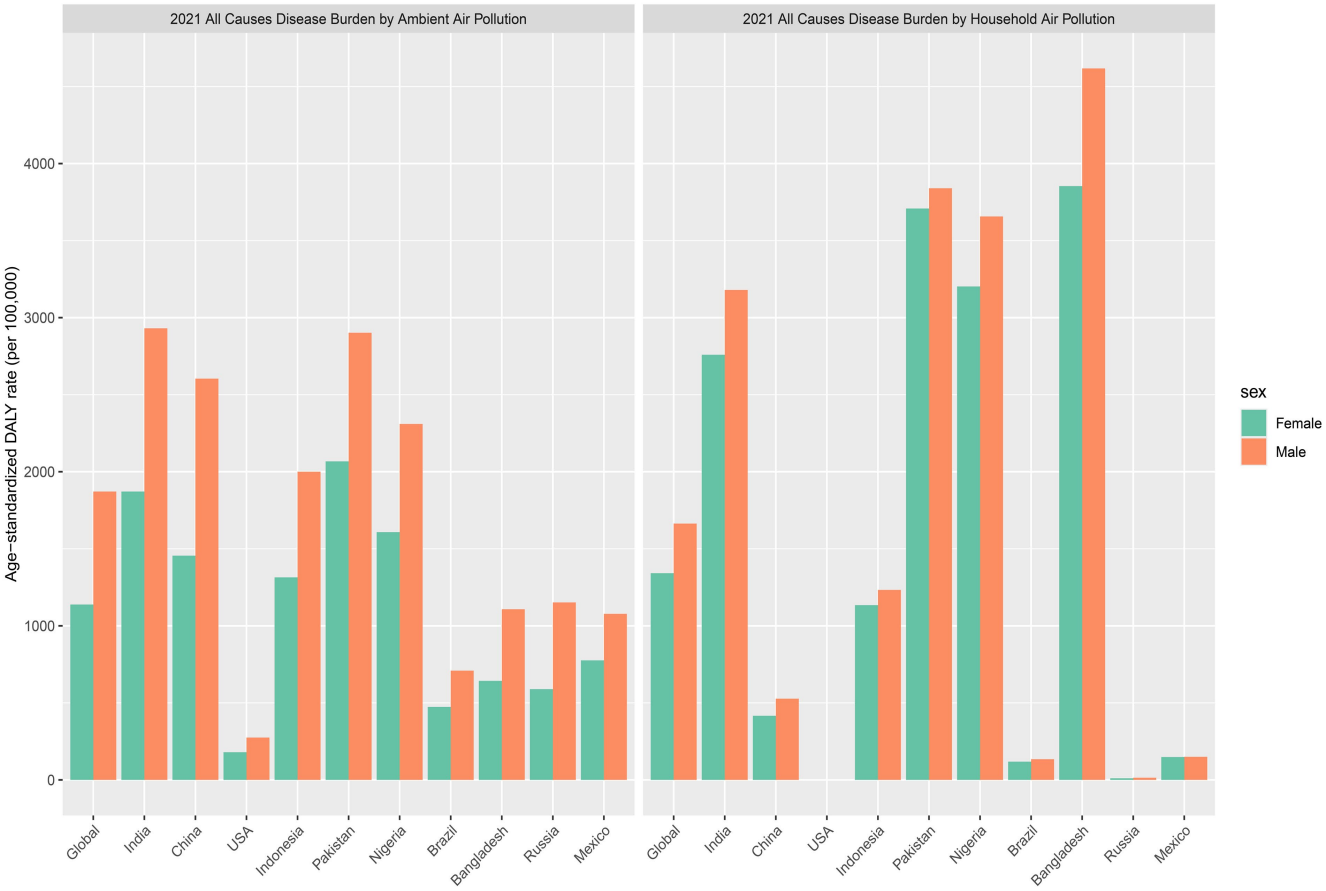
Sex-specific age-standardized DALY rates attributed to APM and HAP, globally and in the ten most populous countries in 2021. DALY, disability-adjusted life year; APM, ambient particulate matter; HAP, household air pollution.

For HAP, the global age-standardized DALY rate in 2021 was 1663.57 per 100,000 for males and 1341.48 per 100,000 for females. India showed rates of 3179.94 and 2759.31; Pakistan, 3839.84 and 3708.47; Nigeria, 3656.88 and 3202.53; Bangladesh, 4617.45 and 3853.86; and Indonesia, 1232.95 and 1134.28, respectively. In contrast, China had markedly lower HAP-related burdens (527.08 for males and 416.22 for females), while the United States reported negligible values for both sexes (approximately 0.14 per 100,000) (Figure 3).

### 3.3 Proportional disease burden attributable to key diseases (2021)

The disease burden attributable to APM and HAP is distributed across seven primary diseases, with varying proportions in different countries.

**Figure 4:**
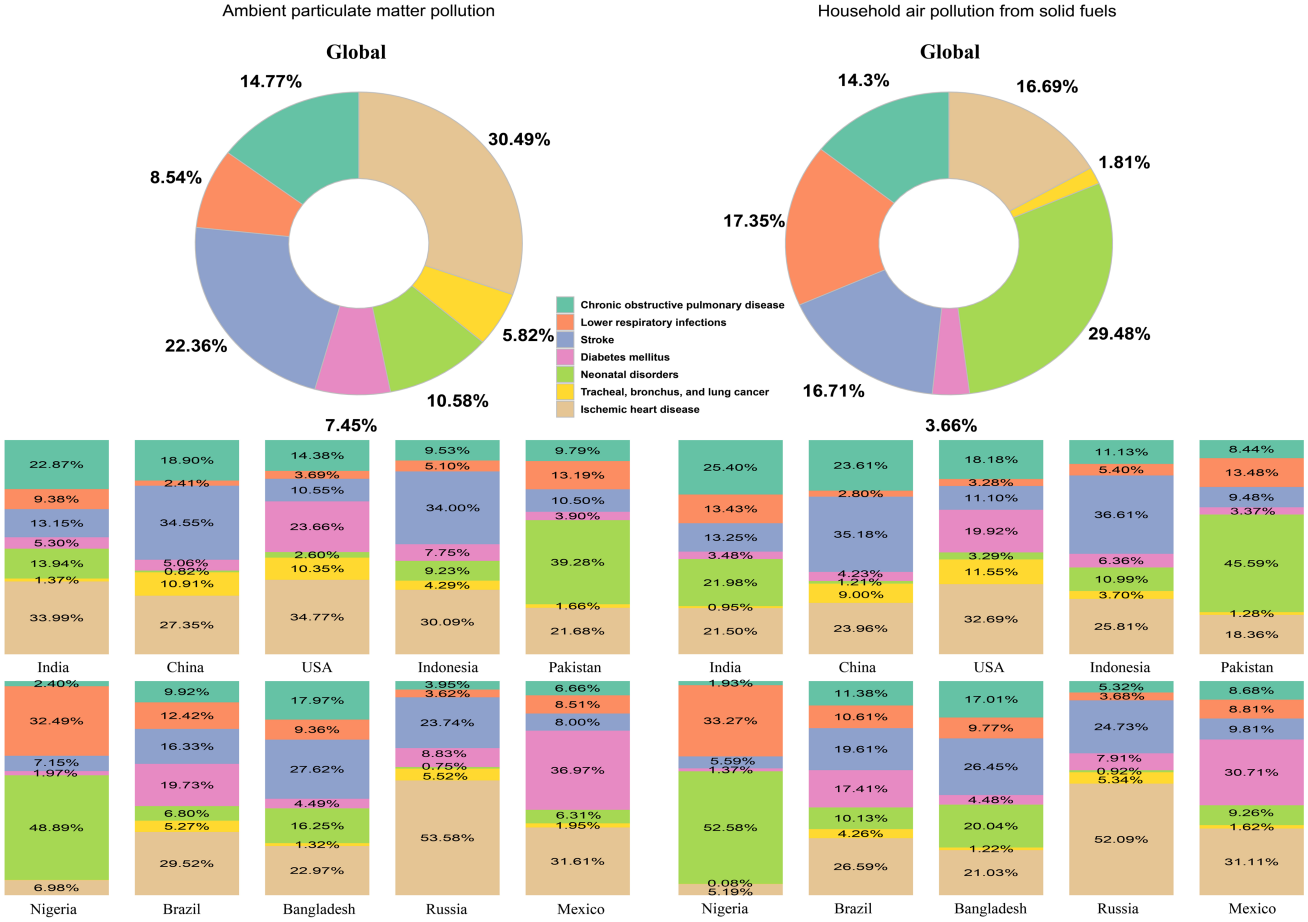
Proportions of DALY counts attributed to APM and HAP across key disease categories in 2021, presented globally and in the ten most populous countries. DALY, disability-adjusted life year; APM, ambient particulate matter; HAP, household air pollution.

Globally, IHD accounted for the largest proportion of the APM-related disease burden, contributing 30.49% of the total burden, followed by stroke (22.36%) and COPD at 14.77%. Neonatal disorders and DM contributed 10.58 and 7.45%, respectively, while Tracheal, Bronchus, and Lung Cancer contributed 5.82%. LRI accounted for 8.54% of the burden. In countries like India, IHD was the leading contributor, with a proportion of 33.99%, followed by COPD at 22.87% and neonatal disorders at 13.94%. In China, stroke contributed the highest proportion at 34.55%, with IHD accounting for 27.35%. The United States displayed a relatively balanced distribution, with IHD contributing 34.77% and stroke contributing 10.55% (Figure 4).

For HAP, neonatal disorders were the largest contributor globally, with a proportion of 29.48%, followed by IHD (16.69%) and stroke (16.71%). COPD contributed 14.3%, and LRI accounted for 17.35%. DM and Tracheal, Bronchus, and Lung Cancer made smaller contributions at 3.66 and 1.81%, respectively. In India, neonatal disorders contributed the highest proportion (21.98%), followed by COPD (25.4%) and IHD (21.5%). In contrast, in China, stroke (35.18%) was the largest contributor, followed by COPD (23.61%) and IHD (23.96%). In the United States, the burden was heavily skewed toward IHD (32.69%) and lower respiratory infections at 3.28%, reflecting the lower HAP burden.

### 3.4 Relationship between socio-demographic index and air pollution-related disease burden

**Figure 5:**
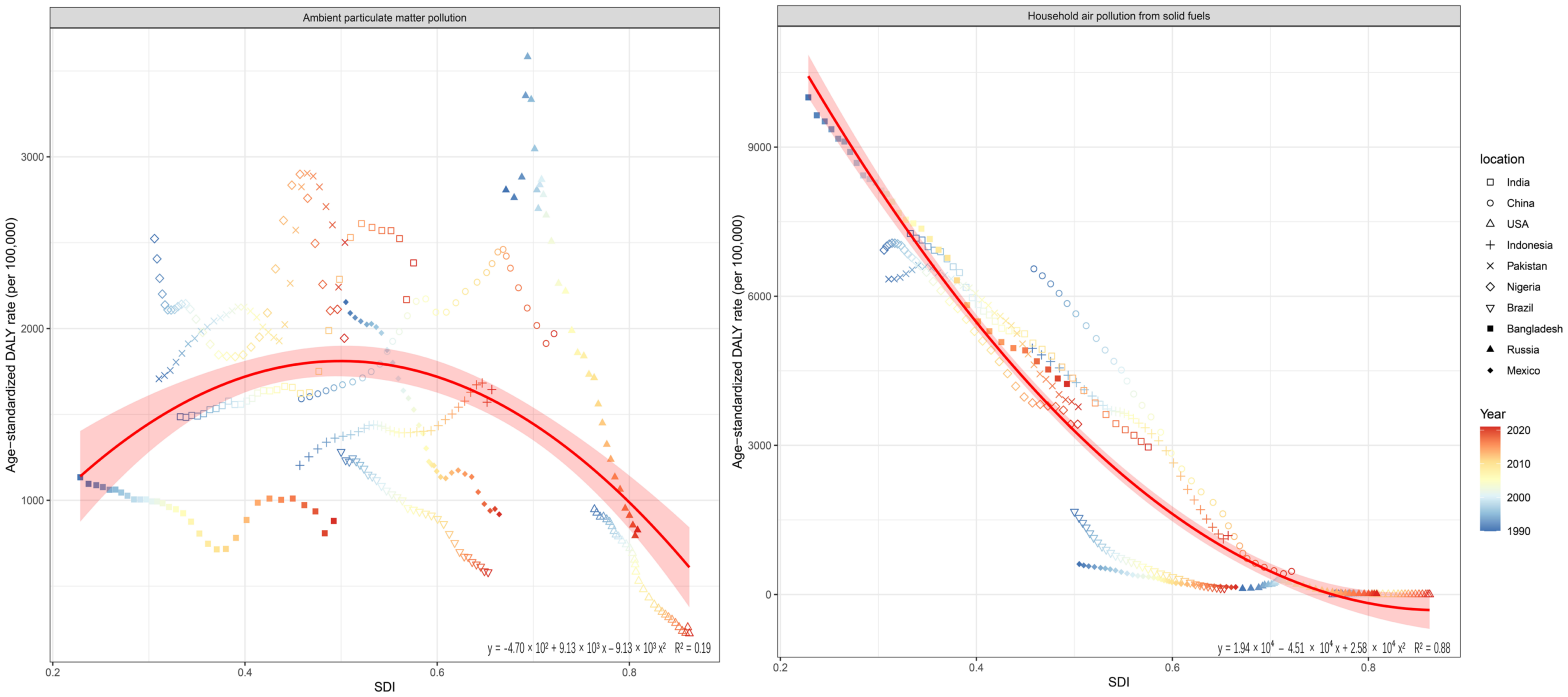
Associations between Socio-demographic Index (SDI) and all-cause age-standardized DALY rates attributed to APM and HAP from 1990 to 2021. DALY, disability-adjusted life year; APM, ambient particulate matter; HAP, household air pollution; SDI, Socio-demographic Index.

Quadratic regression analyses were conducted to assess the association between the SDI and ASDR attributable to APM and HAP. For APM, the fitted quadratic regression equation was y = −4.70 × 10^2^ + 9.13 × 10^3^ x – 9.13 × 10^3^ x^2^, with an *R*^2^ value of 0.19, indicating a relatively limited explanatory capacity of SDI regarding variations in APM-related disease burdens. For HAP, the regression resulted in the equation y = 1.94 × 10^4^–4.51 × 10^4^ x + 2.58 × 10^4^ x^2^, with a higher R^2^ of 0.88, indicating a stronger association between SDI and variations in HAP-related disease burdens (Figure 5).

## 4 Discussion

Across the ten most populous countries analyzed, the disease burden attributable to APM and HAP presents distinct trends shaped by national policies, industrialization, urbanization, and energy transitions. Countries such as the United States have substantially reduced their APM-related disease burdens, largely due to effective implementation of policies such as the Clean Air Act, combined with significant technological advancements in emissions control (18). Nevertheless, challenges persist, particularly from traffic-related and localized industrial emissions. The health burden attributable to HAP in the United States has become negligible in recent years, reflecting the country’s long-standing transition to clean energy and widespread adoption of modern cooking technologies. China presents a complex scenario characterized by rapid industrial growth and urban expansion. While the country has achieved notable progress in reducing HAP through extensive rural energy reforms and the broad dissemination of cleaner household fuels, it continues to face substantial increases in APM-related health burdens, particularly in heavily industrialized urban regions (19). The effectiveness of recent policy initiatives, such as the Air Pollution Prevention and Control Action Plan, underscores the ongoing challenge of balancing rapid economic growth with environmental health protection (20). Countries like Russia, Brazil, and Mexico have seen significant declines in both APM and HAP burdens, largely attributable to economic transitions, urban air quality management programs, and cleaner technology adoption. Russia’s progress reflects substantial changes resulting from the post-Soviet economic restructuring, including industrial modernization and stricter environmental standards (21). Similarly, Brazil and Mexico have improved air quality through focused urban policies and cleaner vehicular emission standards. However, urbanization pressures and rising transportation demands continue to pose significant public health challenges that require sustained policy intervention. India, Pakistan, Indonesia, Bangladesh, and Nigeria continue to face substantial and rising disease burdens from APM, driven primarily by rapid urbanization, industrial expansion, increased vehicle use, and persistent reliance on fossil fuels (22–25). These countries experience difficulties in controlling ambient pollution due to ongoing industrialization pressures and limited effectiveness of environmental regulations. Although HAP has steadily declined in these countries, particularly as cleaner fuels become more accessible, the burden remains elevated due to widespread reliance on biomass and coal for cooking and heating, especially in rural and underserved urban communities (26). Socioeconomic barriers, including affordability of cleaner technologies and limited infrastructure, present significant obstacles to faster progress in reducing these health risks.

Age-specific disparities in disease burden attributable to air pollution are clearly evident, particularly among the youngest and oldest population segments. Children under 5 years are especially vulnerable to HAP, primarily due to their immature respiratory and immune systems. High DALY rates among this group were observed in countries such as Nigeria, Pakistan, Bangladesh, and India. In low-income households, reliance on solid fuels for cooking and heating leads to prolonged exposure to indoor air pollutants, which significantly increases the risk of acute lower respiratory infections and developmental impairments during early childhood (27). APM also contributes to adverse respiratory effects in young children, especially in densely populated urban settings. In contrast, older adults—particularly those aged 70 years and above—exhibit elevated disease burdens mainly due to long-term cumulative exposure to APM. The aging process is accompanied by diminished pulmonary and cardiovascular resilience, making older adult individuals more susceptible to the chronic effects of APM. Long-term inhalation of fine particulate matter accelerates systemic inflammation, oxidative stress, and atherosclerosis, thereby increasing the risk of COPD, ischemic heart disease, and stroke. Countries such as China and Russia demonstrate this trend prominently, due to both rapidly aging populations and a history of industrial emissions (28). To address these disparities, age-targeted and source-specific interventions are necessary. For young children, especially in low- and middle-income countries, priority should be given to reducing HAP exposure through clean cooking initiatives, improved stove technologies, better ventilation, and targeted health education programs. For the older adult, strategies should focus on mitigating ambient air pollution exposure, including enforcing stricter emission controls, expanding green urban infrastructure, enhancing early detection of pollution-related diseases, and increasing access to preventive healthcare services. Strengthening national air quality standards and tailoring interventions to the needs of vulnerable age groups are critical for reducing the unequal burden of air pollution.

Gender differences in air pollution-associated health outcomes are shaped by differential exposures driven by occupational roles, socio-cultural practices, and underlying patterns of disease prevalence. Men generally experience higher burdens from APM due to greater involvement in outdoor occupational activities, including construction, transportation, agriculture, and industrial sectors, resulting in increased pollutant exposure (29). This trend is particularly evident in countries such as India, Pakistan, and China, where male-dominated employment in industries with poor air-quality control significantly contributes to men’s higher health burden. For HAP, although women traditionally bear higher direct exposures due to their involvement in cooking and fuel management tasks, recent evidence indicates that men have a slightly higher overall health burden attributable to HAP. This seemingly counterintuitive finding arises primarily from men’s elevated baseline prevalence of cardiometabolic diseases, such as ischemic heart disease and stroke, which substantially increase their vulnerability to fine particulate matter exposure (30). Thus, while continued efforts to provide clean cooking solutions and improve household ventilation remain essential for protecting women’s health, interventions must also address the broader cardiometabolic risks affecting men to effectively mitigate gender disparities in air pollution-related health outcomes.

The analysis of the proportional disease burden attributable to APM and HAP reveals notable variations across different health outcomes, emphasizing the multifaceted nature of the impacts of air pollution. For APM, IHD emerged as the leading contributor to the disease burden, particularly in countries with high cardiovascular risk factors such as India and China. This is consistent with previous studies highlighting that long-term exposure to fine particulate matter exacerbates cardiovascular diseases through mechanisms such as systemic inflammation and oxidative stress (31). The high burden of stroke, which follows IHD in the APM-related disease profile, also aligns with the known association between particulate matter exposure and cerebrovascular risks (32). In contrast, the burden of disease from HAP is notably higher for neonatal disorders, particularly in low-income countries such as Nigeria, India, and Pakistan. The disproportionate impact of HAP on children under 5 years of age is largely due to their heightened vulnerability to indoor air pollutants, which can lead to conditions such as low birth weight, respiratory infections, and developmental delays (33). Furthermore, the burden of LRI is considerably influenced by HAP. These findings reflect the broader implications of HAP, particularly in regions where solid fuels are still commonly used for cooking, despite ongoing efforts to transition to cleaner cooking technologies (34).

The contrasting associations identified between SDI and disease burdens from APM and HAP provide critical insights for public health and environmental policy. The weak relationship observed between SDI and APM-related health impacts suggests that ambient air pollution exposure is influenced by multifaceted determinants beyond socioeconomic development alone, including factors such as industrial policy effectiveness, urban planning practices, and geographic conditions (35). This complexity has also been noted in previous studies indicating that ambient air pollution does not follow a straightforward socioeconomic gradient due to the interplay of industrial growth and environmental control measures (36). In contrast, the strong quadratic relationship between SDI and HAP underscores a clearer developmental pathway, reflecting significant improvements in indoor air quality as countries progress economically and socially. Higher SDI countries generally have greater resources and policies promoting cleaner household fuels, effective cookstove programs, and improved ventilation, significantly reducing indoor air pollution exposure. This aligns well with established findings highlighting that socioeconomic advancements, including enhanced energy infrastructure and expanded clean cooking solutions, play essential roles in reducing HAP burdens, especially in low- and middle-income countries (37, 38). These findings highlight the necessity for tailored, source-specific interventions addressing different types of air pollution. Policies targeting ambient particulate pollution should incorporate comprehensive urban and industrial regulations, while interventions for HAP must emphasize socioeconomic investments and clean energy transitions. Future research should further explore specific mechanisms through which socioeconomic development can most effectively mitigate air pollution exposures in different national contexts.

This study has several limitations. First, the reliance on secondary data from the GBD Study means that the findings are dependent on the accuracy and completeness of the underlying estimates, which may be less reliable in some low- and middle-income countries. Second, although the ten selected countries represent over half of the global population and encompass diverse geographic and socioeconomic contexts, the limited number of countries may reduce the generalizability of certain findings, particularly regarding the association between SDI and disease burden. Third, the use of SDI as the sole socioeconomic indicator overlooks other relevant factors, such as national environmental policies, healthcare infrastructure, and urban development, which could also influence air pollution exposure and related health outcomes. Fourth, the quadratic regression model employed may oversimplify the complex and potentially nonlinear relationships between SDI and disease burden, as it does not capture local variations in public health capacity or environmental governance. Lastly, this study primarily focuses on long-term trends and does not account for short-term fluctuations in air pollution levels or the immediate impact of recent policy interventions. Future research should aim to incorporate more granular data, a broader range of countries, and a wider array of socio-environmental variables to strengthen the robustness of the findings.

Our findings advocate strongly for intensified policies aligned with the United Nations Sustainable Development Goals (SDGs), particularly Goal 7 (affordable and clean energy) and Goal 3 (good health and well-being). Countries experiencing high disease burdens must prioritize transitioning to clean energy sources, improving air quality regulations, and expanding healthcare infrastructure. Gender-sensitive and age-specific interventions, particularly targeting vulnerable groups like children and the older adult, are crucial. Future research should explore localized socio-economic determinants more deeply, enhancing understanding of how specific policies impact air quality and associated health outcomes. Additionally, interdisciplinary approaches integrating environmental science, public health, and policy analysis will be vital to effectively reduce global air pollution-related health burdens.

## 5 Conclusion

This analysis of air pollution-related disease burdens across the ten most populous countries highlights persistent health disparities linked to APM and HAP. While significant global progress has been made in reducing HAP through cleaner fuels, ambient particulate pollution continues to escalate, particularly in rapidly industrializing countries like India. The burden of APM remains high, and despite some efforts in improving air quality, the overall health risks continue to pose a significant challenge in many countries. Key demographic disparities include heightened risks among older adults from chronic exposure to ambient pollution and significant health impacts on children due to HAP, particularly in lower-income regions. Gender-specific vulnerabilities further complicate these burdens, with occupational exposure for males and household responsibilities for females playing a key role in countries with limited access to cleaner technologies. Addressing these health challenges requires comprehensive, country-specific strategies. Prioritizing clean energy transitions, enhancing environmental regulations, improving household air quality, and ensuring equitable healthcare access are critical steps. Continued policy commitment, alongside targeted interventions that address specific demographic vulnerabilities, will be essential to reducing global health inequalities and advancing progress toward achieving the Sustainable Development Goals.

## Data Availability

The original contributions presented in the study are included in the article/supplementary material, further inquiries can be directed to the corresponding author.

## Footnotes

^1^ https://vizhub.healthdata.org/gbd-results/
